# Aberrant *UBR4* expressions in Hirschsprung disease patients

**DOI:** 10.1186/s12887-019-1879-7

**Published:** 2019-12-12

**Authors:** Gunadi, Alvin Santoso Kalim, Estelita Liana, Aditya Rifqi Fauzi, Dian Nirmala Sirait, Dwiki Afandy, Sagita Mega Sekar Kencana, Eko Purnomo, Kristy Iskandar, Akhmad Makhmudi

**Affiliations:** 1 Pediatric Surgery Division, Department of Surgery/Genetics Working Group, Faculty of Medicine, Public Health and Nursing, Universitas Gadjah Mada/Dr. Sardjito Hospital, Yogyakarta, 55281 Indonesia; 2 Genetics Working Group, Faculty of Medicine, Public Health and Nursing, Universitas Gadjah Mada, Yogyakarta, 55281 Indonesia; 3 Pediatric Surgery Division, Department of Surgery, Faculty of Medicine, Public Health and Nursing, Universitas Gadjah Mada/Dr. Sardjito Hospital, Yogyakarta, 55281 Indonesia; 4 Pediatric Surgery Division, Department of Surgery/Genetics Working Group, Faculty of Medicine, Public Health and Nursing, Universitas Gadjah Mada/UGM Academic Hospital, Yogyakarta, 55291 Indonesia; 5 Department of Child Health/Genetics Working Group, Faculty of Medicine, Public Health and Nursing, Universitas Gadjah Mada/UGM Academic Hospital, Yogyakarta, 55291 Indonesia

**Keywords:** Aberrant expression, Ca^2+^ signaling, Hirschsprung disease, Indonesia, Pathogenesis, *UBR4*

## Abstract

**Background:**

Recently, pathogenic alleles within ubiquitin N-recognin domain-containing E3 ligase 4 (*UBR4*) gene have been shown to be associated with Hirschsprung disease (HSCR). We determined the *UBR4* expressions in Indonesian HSCR patients.

**Methods:**

We analyzed the *UBR4* expressions in the colons of HSCR patient and anorectal malformation (ARM) patient as control by real-time polymerase chain reaction (qPCR).

**Results:**

Thirty-seven patients with non-syndromic HSCR and eighteen controls were involved in this study. qPCR revealed that the *UBR4* expression was strongly decreased (0.77-fold) in the ganglionic group of patients with HSCR compared to the control group with ARM (**ΔC**_**T**_ 2.43 ± 0.36 vs. 2.05 ± 0.69; *p* = 0.009), whereas the *UBR4* expression was also significantly reduced (0.79-fold) in the aganglionic group of patients with HSCR compared to the control group with ARM (**ΔC**_**T**_ 2.39 ± 0.46 vs. 2.05 ± 0.69; *p* = 0.044). However, the *UBR4* expression change was not associated with gender (*p* = 0.35 and 0.80), nor with degree of aganglionosis both in ganglionic and aganglionic colons (*p* = 0.72 and 0.73), respectively.

**Conclusion:**

Our study demonstrates that expression of *UBR4* is decreased in both aganglionic and ganglionic colon of HSCR patients.

## Background

Hirschsprung disease (HSCR) is a multifactorial disease characterized by the absence of ganglion cells in the bowel, causing a functional ileus in infants. It is divided into short-aganglionosis, long-aganglionosis, and total colon aganglionosis [1, 2]. Its frequency in Indonesia is higher (3.1:10,000) [3] than other populations [1, 2]. This difference might be associated with the higher risk allele frequency of *RET* rs2435357 and rs2506030 in Indonesia compared with other populations [4, 5].

Ubiquitin N-recognin domain-containing E3 ligase 4 (UBR4) is a ubiquitin ligase protein that interacts with Ca^2+^ bound calmodulin in cytoplasm and might act as a regulator of Ca^2+^, that is released through ITPR1 [6]. Bowel motility is determined by the synchronized activity of enteric nervous system (ENS), extrinsic nerves, immune cells, interstitial cells of Cajal (ICCs), and smooth muscle cells (SMCs) [7]. ICCs are essential to generate and propagate the electrical cyclical activity (slow waves) in the intestines. The slow waves are transferred into the SMCs to make it depolarize cyclically, resulting in activation of calcium entry and intestines’ contraction [7]. In addition, previous study showed that *UBR4* is one of the novel HSCR genes with an excess of pathogenic alleles in HSCR patients and is expressed in the developing human and mouse fetal gut [8]. Also, there is significant loss of enteric neuronal precursors after *ubr4*-knockdown in zebrafish embryos [8]. Therefore, we determined the *UBR4* expressions in Indonesian HSCR patients with the hypothesis of the *UBR4* expressions decrease in the colon of patients with HSCR.

## Material and methods

### Patients

We involved HSCR patients who underwent pull-through from December 2014 until May 2019 at Dr. Sardjito Hospital, Indonesia [9]. Their parents gave a signed informed consent before joining the study.

We obtained the ganglionic and aganglionic colon of HSCR patients during a pull-through and control colons during a stoma closure from anorectal malformation patients [9].

The Institutional Review Board (IRB) of the Faculty of Medicine, Public Health and Nursing, Universitas Gadjah Mada/Dr. Sardjito Hospital, approved the study (KE/FK/1105/EC/2018).

### Real-time polymerase chain reaction (qPCR)

Total RNA was obtained from HSCR patients and control colons according to our previous study [9], followed by a qPCR to determine the *UBR4* expression using the following primer sets: 5′- TGGACACTCAGCTCACCAAG-3′ (forward) and 5′-GTTCCATCTTGACGCTCCTC-3′ (reverse) [10]. *Glyceraldehyde-3-phosphate dehydrogenase (GAPDH)* was employed as a reference gene for analysis of *UBR4* expression. We used the Livak method to compare the *UBR4* expressions between HSCR patients and control colons [9, 11].

### Statistics

Data was provided as mean ± standard deviation (SD), median (interquartile range, IQR), or frequency. We utilized t-test to determine the significant differences of *UBR4* expression between the ganglionic, aganglionic, and control colon group. We determined a significant level by *p*-value of < 0.05.

## Results

### Baseline characteristics

We involved 37 non-syndromic sporadic HSCR patients and 18 controls. Our patients revealed short-aganglionosis (70%) and long-aganglionosis (30%). Almost half of patients (46%) had transanal endorectal pull-through (46%). The median age at HSCR diagnosis was 4 (IQR, 1–34) months (Table 1).

**Table 1:** Baseline characteristics of HSCR patients in Dr. Sardjito Hospital, Indonesia

Characteristics	N (%); median (IQR)
Gender	
▪ Male	26 (70.3)
▪ Female	11 (29.7)
Type of aganglionosis	
▪ Short-segment	29 (78.4)
▪ Long-segment	8 (21.6)
▪ Total colon aganglionosis	0
Age at HSCR diagnosis (months)	4 (1–34)
Age at definitive surgery (months)	6 (2–30)
Definitive surgery	
▪ Transanal endorectal pull-through	17 (46)
▪ Transabdominal Soave	13 (35)
▪ Duhamel	7 (19)

*HSCR* Hirschsprung disease, *IQR* interquartile range

### UBR4 expressions in HSCR patients

**Fig. 1:**
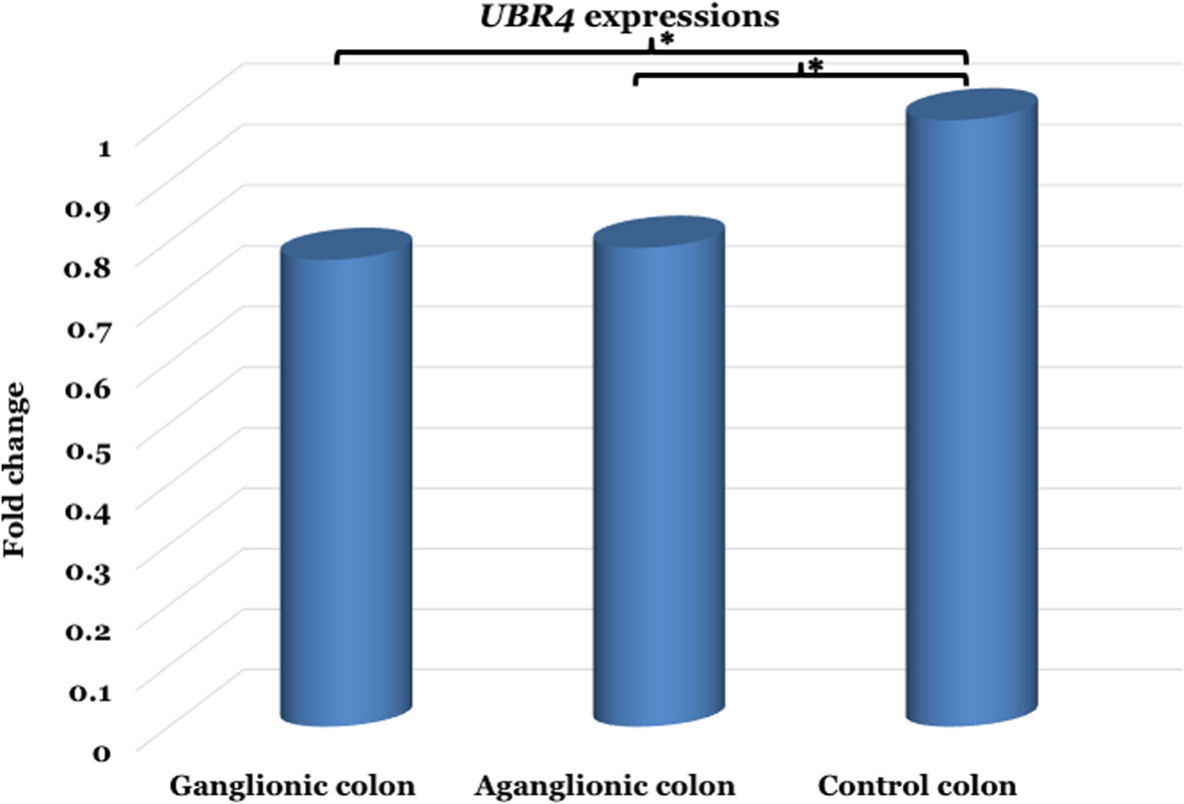
The *UBR4* expression was significantly down-regulated (0.77-fold) in the ganglionic colon group compared to the control group (*p* = 0.009), whereas the *UBR4* expression was also significantly decreased (0.79-fold) in the aganglionic colon group compared to the control group (*p* = 0.044). *, *p* < 0.05 is considered statistically significant

qPCR revealed that the expression of *UBR4* was strongly decreased (0.77-fold) in the ganglionic compared to the control group (**ΔC**_**T**_ 2.43 ± 0.36 vs. 2.05 ± 0.69; *p* = 0.009), whereas the *UBR4* expression was also significantly reduced (0.79-fold) in the aganglionic compared to the control group (**ΔC**_**T**_ 2.39 ± 0.46 vs. 2.05 ± 0.69; *p* = 0.044) (Table 2 and Fig. 1).

**Table 2:** The *UBR4* expressions in the HSCR patients and control colons

	*UBR4* (ΔC_T_ ± SD)	ΔΔC_T_ (95% CI)	Fold change (2^-ΔΔC^_T_)	*p*-value
Ganglionic colon	2.43 ± 0.36	0.39 (0.10–0.67)	0.77	0.009*
Aganglionic colon	2.39 ± 0.46	0.34 (0.01–0.67)	0.79	0.044*
Control colon	2.05 ± 0.69			

*, *p* < 0.05 is considered statistically significant; HSCR, Hirschsprung disease

Next, we compared the *UBR4* expressions between ganglionic and aganglionic colon group. qPCR showed that the *UBR4* expressions were not significantly different between two groups (**ΔC**_**T**_ 2.43 ± 0.36 vs. 2.39 ± 0.46; *p* = 0.64).

### Association between UBR4 expressions and baseline characteristic of HSCR patients

We examined the association between *UBR4* expressions with gender and degree of aganglionosis in HSCR patients in this cohort. The *UBR4* expressions were not significantly associated with gender (*p* = 0.35 and 0.80), nor with type of aganglionosis both in ganglionic and aganglionic colons (*p* = 0.72 and 0.73), respectively (Table 3).

**Table 3:** Association between *UBR4* expressions and baseline characteristics of HSCR patients

*UBR4*	Male (*n* = 26) (ΔC_T_ ± SD)	Female (*n* = 11) (ΔC_T_ ± SD)	ΔΔC_T_ (95% CI)	Fold change (2^-ΔΔC^_T_)	*p*-value
Ganglionic Colon	2.40 ± 0.37	2.52 ± 0.35	−0.12 (−0.38–0.14)	1.09	0.35
Aganglionic Colon	2.38 ± 0.48	2.42 ± 0.45	−0.05 (− 0.41–0.32)	1.03	0.80
	Short-segment (*n* = 29) (ΔC_T_ ± SD)	Long-segment (*n* = 8) (ΔC_T_ ± SD)			
Ganglionic Colon	2.42 ± 0.38	2.48 ± 0.30	− 0.05 (− 0.35–0.24)	1.04	0.72
Aganglionic Colon	2.40 ± 0.46	2.32 ± 0.55	0.08 (− 0.39–0.55)	0.95	0.73

## Discussion

We are able to show for the first time the aberrant *UBR4* expression in HSCR patients. We determined *UBR4* expressions in the aganglionic, ganglionic, and control colons using qPCR. Our study reveals a significant difference of *UBR4* expression between HSCR patients’ colons and control colons, implying that the aberrant *UBR4* expression could be one of the contributing factors of Indonesian HSCR patients.

UBR4 has a role in Ca^2+^ signaling and is involved in neuronal excitability [12] since it interacts with Ca^2+^ bound calmodulin in cytoplasm and acts as a regulator of Ca2+, which is released through ITPR1 [6]. Ca^2+^ signaling is important to maintain the intestines’ motility, together with the synchronized activity of ENS, extrinsic nerves, immune cells, ICCs, and SMCs [7]. The intestines’ contraction is induced by the activation of calcium entry due to cyclically depolarization of SMCs. ICCs generate and propagate the slow waves to be transferred into SMCs [7]. HSCR pathogenesis includes the compromised condition of genes responsible for the ENS development [1, 2, 4, 5, 8], the neurotransmitters expressed by the ENS neurons [13] and/or their interactions. Recently, pathogenic alleles within the *UBR4* gene have been shown to be associated with HSCR [8]. Furthermore, a recent study demonstrated that the death of *Ubr4*-deficient mice embryos was correlated with developmental defects in various processes, including neurogenesis, due to failure to preserve cell integrity and adhesion [14]. It has been shown that neurogenesis in embryos is strongly affected by the dysregulation of cell adhesion molecules [15]. Lack of *UBR4* causes the rapid depletion of other cells’ surface proteins as well, such as platelet-derived growth factor receptor (PDGFR) [14]. In addition, previous study revealed that *SK3* is highly expressed in the PDGFRα+ cells [13], which together with ICCs and SMCs regulate intestinal peristalsis [16]. Our results further support the importance of *UBR4* in the HSCR pathogenesis by providing new evidence of the aberrant *UBR4* expressions in HSCR patients’ colons. We hypothesize that the aberrant *UBR4* expressions contribute to the pathogenesis of HSCR in our patients by affecting the expression of *SK3* in the PDGFRα+ cells.

Moreover, our study for the first time demonstrated that the decreased *UBR4* expression also occurred in the ganglionic colon of HSCR patients. It has been shown that several aberrant gene expressions, including *SK3* [9, 17], *Cx26* and *Cx43* [18], and *NOS* [19], were significantly associated with the persistent intestinal symptoms in HSCR patients after an appropriately completed surgery. Whether the aberrant *UBR4* expression in the ganglionic colon is also correlated with the persistence of bowel symptoms after pull-through in HSCR patients warrants further investigation.

It should be noted that our study used ARM patient colon as control. To the best of our knowledge, there is no study comparing the *UBR4* expression between ARM patient colon and other colonic specimens. These facts should be considered during the interpretation of our findings since most ARM patients also show the intestinal motility problem [20]. Therefore, further analysis using controls without any bowel motility problem is needed to confirm our results.

Moreover, future studies are necessary to further confirm the role of *UBR4* in the pathogenesis of HSCR by checking the decreased of UBR4 protein expressions using western blot or immunohistochemistry and screening the pathogenic variant in the *UBR4* gene using sequencing in HSCR patients.

## Conclusion

Our study demonstrates that expression of *UBR4* is decreased in both aganglionic and ganglionic colon of HSCR patients.

## Data Availability

All data generated during this study are contained in the submission. The raw data are available from the corresponding author on reasonable request.

## Abbreviations

ENS: Enteric nervous system
GAPDH: Glyceraldehyde-3-phosphate dehydrogenase
HSCR: Hirschsprung disease
ICCs: Interstitial cells of Cajal
qPCR: Quantitative real-time polymerase chain reaction
SMCs: Smooth muscle cells
UBR4: Ubiquitin N-recognin domain-containing E3 ligase 4
